# Proprotein Convertase Subtilisin/Kexin Type 9 (PCSK9) Can Mediate Degradation of the Low Density Lipoprotein Receptor-Related Protein 1 (LRP-1)

**DOI:** 10.1371/journal.pone.0064145

**Published:** 2013-05-13

**Authors:** Maryssa Canuel, Xiaowei Sun, Marie-Claude Asselin, Eustache Paramithiotis, Annik Prat, Nabil G. Seidah

**Affiliations:** 1 Laboratory of Biochemical Neuroendocrinology, Clinical Research Institute of Montreal, affiliated to the University of Montreal, Montreal, Quebec, Canada; 2 Caprion Proteomics Inc, Montreal, Quebec, Canada; Tohoku University, Japan

## Abstract

Elevated LDL-cholesterol (LDLc) levels are a major risk factor for cardiovascular disease and atherosclerosis. LDLc is cleared from circulation by the LDL receptor (LDLR). Proprotein convertase subtilisin/kexin 9 (PCSK9) enhances the degradation of the LDLR in endosomes/lysosomes, resulting in increased circulating LDLc. PCSK9 can also mediate the degradation of LDLR lacking its cytosolic tail, suggesting the presence of as yet undefined lysosomal-targeting factor(s). Herein, we confirm this, and also eliminate a role for the transmembrane-domain of the LDLR in mediating its PCSK9-induced internalization and degradation. Recent findings from our laboratory also suggest a role for PCSK9 in enhancing tumor metastasis. We show herein that while the LDLR is insensitive to PCSK9 in murine B16F1 melanoma cells, PCSK9 is able to induce degradation of the low density lipoprotein receptor-related protein 1 (LRP-1), suggesting distinct targeting mechanisms for these receptors. Furthermore, PCSK9 is still capable of acting upon the LDLR in CHO 13-5-1 cells lacking LRP-1. Conversely, PCSK9 also acts on LRP-1 in the absence of the LDLR in CHO-A7 cells, where re-introduction of the LDLR leads to reduced PCSK9-mediated degradation of LRP-1. Thus, while PCSK9 is capable of inducing degradation of LRP-1, the latter is not an essential factor for LDLR regulation, but the LDLR effectively competes with LRP-1 for PCSK9 activity. Identification of PCSK9 targets should allow a better understanding of the consequences of PCSK9 inhibition for lowering LDLc and tumor metastasis.

## Introduction

Elevated plasma cholesterol levels result in excess cholesterol deposition in arterial vessel walls, and are a major risk factor for atherosclerosis and premature death by coronary artery disease [1]. In the blood, cholesterol is transported in lipoprotein particles, ∼70% of which in humans are low-density lipoproteins (LDL). LDL is constantly cleared by internalization into cells by the LDL receptor (LDLR) [2], [3]. The proprotein convertase subtilisin/kexin 9 (PCSK9) enhances the degradation of the LDLR, and is well-established as a gene associated with familial hypercholesterolemia, along with *LDLR, APOB* [2]–[4] and very recently *APOE* [5]. By an as yet unknown mechanism(s), and independent of its enzymatic activity [6], PCSK9 enhances the degradation of cell surface LDLR [7]–[9] in endosomes/lysosomes [10], resulting in increased circulating LDL cholesterol (LDLc). In fact, *Pcsk9* ***^−/−^*** mice exhibit higher levels of LDLR in liver and ∼42% less circulating total cholesterol, with a ∼80% drop in LDLc [11], [12], emphasizing the therapeutic potential of a PCSK9 inhibitor/silencer [13].

PCSK9, which is synthesized [14] and secreted [12] primarily from hepatocytes, is comprised of a signal peptide (amino acid, aa 1–30), a prosegment (Pro; aa 31–152), a catalytic domain (aa 153–407), a hinge region (aa 408–452) and a C-terminal Cys-His-rich domain (CHRD; aa 453–692) [15]. Following translocation into the endoplasmic reticulum, the prosegment is autocatalytically cleaved at the VFAQ_152_↓SIP site [8], [14]. PCSK9 is secreted as a stable, enzymatically inactive, non-covalent complex [Pro.PCSK9] [8], [14], [16]. At the cell surface, secreted PCSK9 binds at neutral pH to the EGF-A-like repeat of the LDLR *via* its catalytic domain [17], [18]. While this interaction is sufficient for internalization of the [LDLR.PCSK9] complex, the ability of PCSK9 to induce lysosomal degradation of the LDLR requires the presence of its CHRD [19]. It was proposed that the CHRD can *in vitro* associate with the ligand binding domains of the LDLR, especially at acidic pHs [20]. PCSK9 can induce the degradation of the LDLR either via an intracellular or extracellular pathway, the latter requiring secretion of PCSK9 and internalization of the cell surface [LDLR.PCSK9] complex into clathrin–coated pits [21]. While the integrity of the CHRD is essential for the extracellular pathway [19], the loss of an internal M2-domain of the CHRD does not affect the intracellular pathway [22], emphasising some of the differences between these sorting pathways.

The activity of PCSK9 on cell surface LDLR, and on its internalization in particular, have been demonstrated to also require the autosomal recessive hypercholesterolemia (ARH) adaptor protein [23]. ARH binds the **NP**V**Y** motif in the cytosolic tail (CT) of the LDLR, the β2-adaptin subunit of AP-2, and the clathrin heavy chain, thereby recruiting the receptor into clathrin-coated pits [24], [25]. The importance of the **NP**X**Y** motif is illustrated by a naturally occurring mutation in which the tyrosine is mutated into a cysteine (Y807C), thereby preventing receptor clustering and internalization [26]. When primary hepatocytes were isolated from livers of *Arh* ***^−/−^*** mice and treated with up to 10 μg/ml of purified PCSK9, Western blot analysis revealed no change in total or cell surface LDLR levels [23], emphasizing the importance of ARH in the mechanism of PCSK9-induced LDLR degradation. However, a recent study investigating the role of the CT of the LDLR demonstrated that an early termination LDLR mutant (K811X), which lacks its CT (ΔCT), was still degraded in CHO cells treated exogenously with the PCSK9 gain-of-function (GOF) mutant D374Y (PCSK9^D374Y^) [27], [28]. CHO cells expressing the ΔCT construct maintained their capacity to uptake LDL and internalize PCSK9 [28]. Given the requirement for ARH and the ability of PCSK9 to act on the LDLR in the absence of the receptor's CT, these findings would suggest the presence of an additional factor(s) at the cell surface, which potentially interacts with either the LDLR, PCSK9, or both to mediate the internalization and/or degradation of the [LDLR.PCSK9] complex.

Herein, our objective was to identify and investigate novel partners implicated in the PCSK9-regulated trafficking of the LDLR. This led us to identify the low density lipoprotein receptor-related protein 1 (LRP-1) as a receptor whose degradation is induced by PCSK9 in two melanoma cell lines, in which we previously showed that the lack of host mouse PCSK9 reduced their metastasis in liver [29], [30]. We therefore investigated the ability of PCSK9 to modulate LRP-1 protein levels and the possibility that PCSK9 may modulate differentially LRP-1 from the LDLR, and/or represent one of the sought co-factors in the PCSK9-induced LDLR degradation.

## Materials and Methods

### Cell Culture and Transfection

HEK293, HepG2, and B16F1/F10 (ATCC) cell lines were grown in Dulbecco's modified Eagle's medium with 10% fetal bovine serum (Invitrogen), whereas CHO-K1, CHO-A7 and CHO 13-5-1 (a generous gift from David J. Fitzgerald, laboratory of Molecular Biology, NIH, USA) cells were grown in Ham's F-12 medium/Dulbecco's modified Eagle's medium (50∶50) supplemented with 10% fetal bovine serum. Cells were maintained at 37°C under 5% CO_2_. Stable PCSK9-shRNA transfectants obtained in HepG2 from Robert Day (University of Sherbrooke, QC, Canada), were previously described [21]. At 80–90% confluence, CHO cell lines were transiently transfected with Lipofectamine 2000 (Invitrogen), whereas HEK293 cells were transfected with jetPRIME (Polyplus) according to the manufacturers' protocols. Conditioned media was produced by replacing cell culture media 24 h subsequent to transfection with serum-free medium. After overnight incubation on the cells, the conditioned media was collected, and some samples were analyzed by ELISA for PCSK9 levels [31], [32] and applied as indicated. Secreted PCSK9 contains mostly full length PCSK9, but also its furin cleaved product, PCSK9-ΔN_218_ [33].

Primary hepatocytes were isolated from the livers of 3 month old WT C57BL/6 mice. Mice were maintained on a chow diet in a 12 h light/12 h dark schedule and used at approximately 3 months of age. Experiments were performed as previously described (24) and in accordance with protocols approved by the bioethics committee of the Clinical Research Institute of Montreal. Primary hepatocytes were cultured overnight in serum-free hepatoZYME medium (Invitrogen).

### Plasmids and antibodies

C-terminally V5-tagged full-length human LDLR, LDLR lacking its CT (ΔCT), and LDLR lacking its CT in which the TMD was replaced with that of angiotensin converting enzyme 2 (ACE2) (ΔCT_TMDace2_) or the very low density lipoprotein receptor (VLDLR) (ΔCT_TMDvldlr_) constructs were cloned into the pIRES2-EGFP vector (Clontech, Mountain View CA). Other cDNAs used included those in the phCMV3 vector, namely PCSK9-LAMP1 and CHRD-LAMP1 which lacks the catalytic domain of PCSK9. These chimeric PCSK9 constructs were coupled to the TMD and CT of LAMP1 with C-terminal V5-tags, as previously reported [34]. C-terminal V5-tagged wild-type human PCSK9, and the GOF D374Y mutant (PCSK9^D374Y^) cDNAs were cloned into pIRES2-EGFP [14].

Rabbit polyclonal anti-human PCSK9 antibody was raised in our laboratory as described [10]. PCSK9-V5, its GOF mutant, and chimeric constructs were detected using a mouse monoclonal (mAb) anti-V5 antibody from Invitrogen. The same mAb-V5 was used to detect full-length LDLR-V5 and its truncated chimeric constructs, whereas endogenous LDLR was visualized in HEK293 and HepG2 cells using a goat anti-human LDLR polyclonal antibody purchased from R/D Systems. Mouse anti-LDLR polyclonal antibody was also purchased from R/D Systems. The LDLR in CHO-K1 and CHO 13-5-1 cell lines was detected using an anti-hamster rabbit polyclonal LDLR antibody (BioVision). Rabbit anti-human EGFR antibody was also purchased from BioVision. Endogenous LRP-1 was detected in all cases with a rabbit polyclonal antibody from Abcam recognizing the ∼85 kDa TMD-containing C-terminal LRP-1 β-fragment, while an anti- β-actin antibody was acquired from Sigma.

### qPCR

Quantitative real time PCR analysis (qPCR) of PCSK9, LDLR and LRP-1 RNA preparations from B16F1/10 cells was performed as previously described [35]. Primers used were as follows: LRP-1 (5′-GACCAGGTGTTGGACACAGATG-3′ *versus* 5′-AGTCGTTGTCTCCGTCACACTTC-3′), PCSK9 (5′-CAGGGAGCACATTGCATCC-3′ *versus* 5′-TGCAAAATCAAGGAGCATGGG-3′), LDLR (5′-GGAGATGCACTTGCCATCCT-3′ *versus* 5′-AGGCTGTCCCCCCAAGAC-3′). The Mx3500P system from Stratagene was used to perform and analyze the qPCRs, using the TATA-box binding protein (TBP) as a normaliser.

### Western blot analyses

Cells were washed three times in PBS and lysed on ice in 1x RIPA buffer (50 mm Tris-HCl, pH 8.0, 1% (v/v) Nonidet P-40, 0.5% sodium deoxycholate, 150 mm NaCl, and 0.1% (v/v) SDS) supplemented with 1x complete protease inhibitor mixture (Roche Applied Science). Proteins were separated by 8% SDS-PAGE and transferred overnight to HyBond nitrocellulose membranes (GE Healthcare). The membranes were blocked for 1 h at room temperature in TBS-T (50 mm Tris-HCl, pH 7.5, 150 mm NaCl, 0.1% Tween 20) containing 5% nonfat dry milk. Membranes were incubated with primary antibodies overnight at 4°C in a 5% milk-TBS-T solution at the following dilutions: human and mouse LDLR (1∶1,000), hamster LDLR (1∶5,000), LRP-1 (1∶20,000), mAb-V5 (1∶5,000), PCSK9 (1∶2,500), EGFR (1∶1000), and β-actin (1∶5,000). Appropriate horseradish peroxidase-conjugated secondary antibodies were used at 1∶10,000 in 5% milk-TBS-T and revealed with chemiluminescence using the ECL plus kit (GE Healthcare). Quantification was performed relative to β-actin using the NIH ImageJ software. In all cases at least 3 independent experiments were performed, and representative images and their quantifications are shown.

### FACS

HEK293 cells transfected with LDLR-V5 constructs were incubated overnight at 37°C with PCSK9-V5 or empty pIRES-V5 vector control conditioned media (∼0.7 μg/ml, estimated by ELISA) [31], [32]. The cells were washed three times with calcium/magnesium-free Dulbecco's PBS containing 0.5% bovine serum albumin (Sigma) and 1 g/L glucose (Solution A). Cells were then incubated for 5 min at 37°C with 500 μl of 1×Versene solution (Invitrogen) and layered onto 5 ml of Solution A. Cells were then centrifuged for 5 min at 1,000 rpm and re-suspended in 1 ml of Solution A containing 1∶100 of monoclonal LDLR antibody C7 directed against human LDLR (mAb-C7, Santa Cruz Biotechnology) for 30 min. Cells were washed once with 5 ml of Solution A, centrifuged, and re-suspended for 30 min in 1 ml of PBS containing 1∶250 of Alexa Fluor 647 donkey anti-mouse (Molecular Probes). Cells were washed and re-suspended in 300 μl of PBS 0.2% of propidium iodide. Viable cells (propidium iodide-negative) were then analyzed by fluorescence-associated cell sorting (FACS) for both propidium iodide and Alexa Fluor 647 using the CyAn flow cytometer (Beckman Coulter).

### Proteomics

Livers from WT and Pcsk9−/− mice were homogenized in 3 ml of ice-cold homogenization buffer (0.5 M sucrose 10 mM Tris pH 7.4) using a Potter-Elvehjem homogenizer using 10 up and down strokes with a Teflon pestle driven at 1,200 rpm. The homogenate was adjusted to 1.5 M sucrose using a 2.55 M sucrose solution. A 14 ml SW40Ti ultra-clear centrifuge tube (Beckman Coulter) was layered with homogenate followed by 5 ml of 1.2 M sucrose, and topped with 0.8 M sucrose. The samples were centrifuged for 2 h at 155,000×g at 4°C and light membranes were harvested from the 0.8–1.2 M sucrose interface and stored at −80°C until proteomic analyses. Samples were digested with 10-fold excess of trypsin (Promega) and 450 μl of each sample was loaded onto a 100×2.1 mm SCX Biobasic column (Thermo) using a gradient of ammonium formate over 30 min. Three fractions were collected from 3.9 to 20 min. Each fraction was analyzed using a LTQ Orbitrap XL mass spectrometer (Thermo) coupled to a Nano-Acquity liquid chromatography system (Waters). Components were detected and matched across all samples and compared for relative peak intensity. Peak intensity was normalized to account for small differences in protein concentration between samples. ANOVA was then applied to identify peptides that were differentially expressed between the groups of interest. False detection rate and q-value were calculated based on the p-values obtained from the ANOVA model, using Storey's method to make multiple testing adjustments. Fold-changes between PCSK9 WT and Pcsk9−/− mice were calculated using the parameters determined by the ANOVA model. Protein identification was done using Mascot software (Matrix Science) with the International Protein Index mouse protein sequence database.

## Results

### PCSK9 acts on the LDLR independent of the receptor's CT and TMD

**Figure 1:**
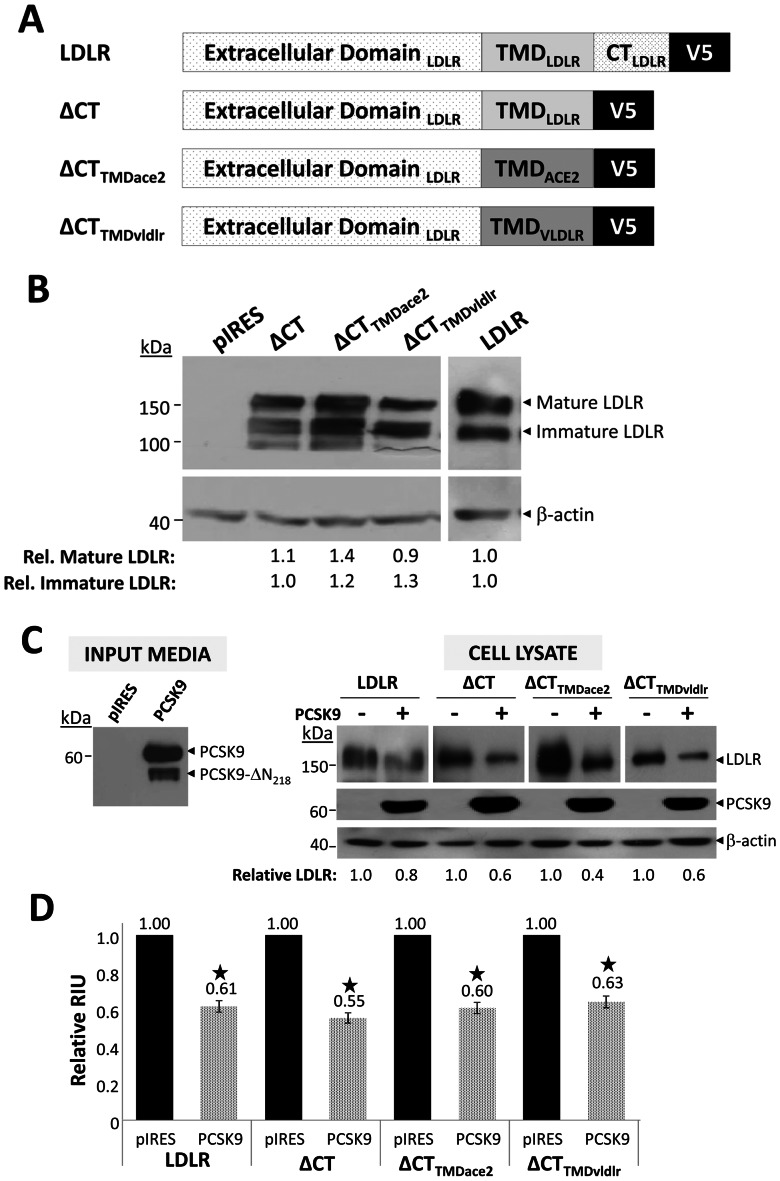
PCSK9 acts on the LDLR independent of the receptor's CT and TMD. **A**) Generation of chimeric truncated LDLR-V5 constructs. Schematic representation of the LDLR, LDLR lacking its CT (ΔCT), and ΔCT in which the LDLR TMD was swapped with that of ACE2 (ΔCT_TMDace2_) or VLDLR (ΔCT_TMDvldlr_). All constructs contained a C-terminal V5-tag. **B**) Expression in HEK293 cells. WT and chimeric LDLR constructs were transfected in HEK293 cells. Construct expression was assessed by immunoblotting with mAb-V5. Both mature and immature forms of the LDLR were detected. β-actin was used as a loading control. **C)** PCSK9 induces LDLR degradation independent of the LDLR's CT and TMD. LDLR, ΔCT, and the ΔCT_TMDace2_ and ΔCT_TMDvldlr_ chimeric constructs were expressed in HEK293 cells. Twenty-four hours post-transfection, the cells were treated overnight with empty vector control pIRES-V5 or PCSK9-V5 conditioned media, which contains both full length PCSK9 and its furin cleaved product at Arg_218_, PCSK9-ΔN_218_ [33]. Cells were lysed in 1x RIPA and subjected to Western blot analysis. LDLR and PCSK9 were detected with mAb-V5. β-actin was employed as a loading control. The ability of PCSK9 to induce degradation of the LDLR constructs was quantified using NIH ImageJ software and calculated relative to treatment with pIRES conditioned media. Data are representative of at least three independent experiments. **D**) PCSK9 reduces cell surface LDLR levels independent of the receptor's CT and TMD. To assess the ability of PCSK9 added exogenously to HEK293 cells expressing the LDLR or its chimeric constructs, transfected cells were treated overnight with empty vector control pIRES-V5 or PCSK9-V5 conditioned media. Subsequently, surface LDLR was quantified by FACS analysis. The values obtained after treatment with PCSK9 are represented graphically relative to treatment with control pIRES. Data are representative of at least three independent experiments. Error bars represent SEM. *, *p*<0.05 (Student's t test).

The ARH adaptor protein is required for exogenous PCSK9 to induce degradation of the LDLR [23]. However, it has more recently been shown that LDLR lacking its CT (ΔCT) is still sensitive to PCSK9 added exogenously to CHO-K1 cells [28]. Without its CT, and hence the **NP**V**Y** motif known to be required for ARH recruitment [24], [25], the ability of exogenous PCSK9 to act on ΔCT suggests that LDLR's transmembrane domain (TMD) may participate in receptor sorting, or that LDLR has a co-receptor. To eliminate the first possibility that the TMD of the LDLR is required for its PCSK9-mediated degradation, we generated two chimeric LDLR constructs, wherein the CT of the LDLR was removed (ΔCT), and the TMD replaced with that of ACE2 or that of the VLDLR (Figure 1A). The TMDs of ACE2 and VLDLR have only 17% and 55% sequence identity to that of the LDLR, respectively. Moreover, while the TMDs of the LDLR and VLDLR are of the same length (20 aa), that of ACE2 is longer (27 aa). The V5-tagged ΔCT and its two chimeric constructs (ΔCT_TMDace2_ and ΔCT_TMDvldlr_) were similarly expressed in HEK293 cells as detected by immunoblotting with mAb-V5 (Figure 1B) and quantified relative to β-actin. Similar to full length LDLR, in the three constructs, both the immature (∼110 kDa) and mature (∼150 kDa) glycosylated forms of the LDLR were detected.

We next determined whether or not the CT and TMD are required for PCSK9 activity on LDLR, ΔCT, ΔCT_TMDace2_ and ΔCT_TMDvldlr_. Accordingly, HEK293 cells transfected with these constructs were incubated with conditioned media of HEK293 cells expressing either a control vector (pIRES) or PCSK9 (∼0.7 μg/ml) [31], [32]. Incubated cells were then lysed and subjected to Western blot analysis (Figure 1C), or collected and cell surface LDLR levels examined by FACS (Figure 1D). Relative to β-actin, using a mAb-V5, Western blot analysis showed that LDLR was reduced by ∼20% in cells treated with PCSK9 (Figure 1C). Similarly, decreases of ∼40–60% were observed in cells expressing ΔCT (also observed in hepatic HuH7 cells, *not shown*), ΔCT_TMDace2_ or ΔCT_TMDvldlr_ (Figure 1C). FACS analysis revealed that regardless of the LDLR construct examined, exogenous PCSK9 resulted in a ∼40–50% decrease in cell surface LDLR levels compared to cells treated with control conditioned media (Figure 1D). Hence, the ability of extracellular PCSK9 to act on the LDLR is not dependent on the receptor's CT or TMD. This finding suggests that there may be a critical [LDLR.PCSK9] interacting protein at the cell surface.

### PCSK9 enhances the degradation of the LRP-1 in melanoma B16 cells more effectively than LDLR

**Figure 2:**
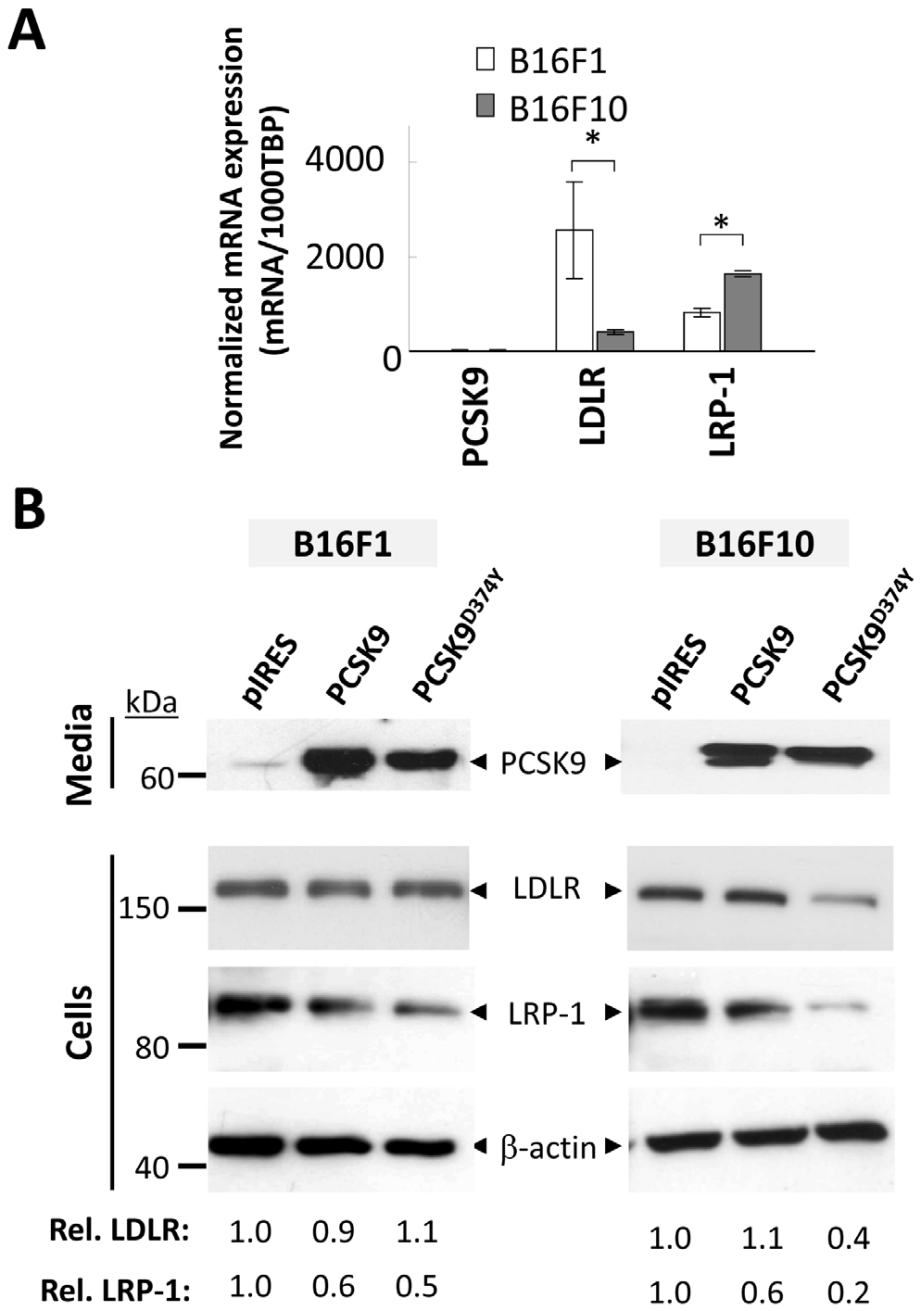
PCSK9 enhances the degradation of the LRP-1 in melanoma B16 cells. **A**) LDLR and LRP-1 are differentially regulated in B16F1 and B16F10 cells. The expression levels of endogenous PCSK9, LDLR, and LRP-1 mRNA were quantified by qPCR in B16F1 and B16F10 melanoma cells. Expression values were normalized to that of housekeeping gene TBP mRNA. Error bars represent SEM. *, *p*<0.05 (Student's t test). **B)** LDLR and LRP-1 are differentially regulated. B16F1 and B16F10 cells were transfected with control empty pIRES vector, PCSK9 or the GOF PCSK9^D374Y^. The media of these cells were analyzed by Western blot to show the expression and secretion of PCSK9 using mAb-V5. The protein levels of LDLR, LRP-1 and β-actin were revealed in total cell lysates using anti-mouse LDLR, anti-LRP-1 and anti-β-actin antibodies. The immunoblots were submitted to quantitative analysis using NIH ImageJ software. The relative intensities were normalized to β-actin and are representative of three independent experiments.

LRP-1 is known to bind more than 40 different ligands [36], [37], and has been implicated in various pathologies including cancer/metastasis [38]. LRP-1 is expressed together with LDLR in low metastatic B16F1 and highly metastatic B16F10 tumour cells (obtained by serial passage of B16F1 cells as lung nodules) [39], both of which do not express PCSK9 mRNA endogenously (Figure 2A). Interestingly, we observed an inverse relationship between the mRNA expression of LDLR and LRP-1 in these cells. Thus, LDLR is expressed 5-fold more in the less aggressive B16F1 cells, and LRP-1 is 2-fold more abundant in the more aggressive B16F10 cells (Figure 2A).

Recently, we observed that PCSK9 enhances the ability of mouse melanoma B16 cells to colonize liver in a metastasis model [29]. Since these cells do not express PCSK9 (Figure 2A), it was possible that exogenous PCSK9 could regulate the levels of LDLR or LRP-1 in B16 cells. Accordingly, we transiently transfected PCSK9 and its GOF D374Y mutant in both B16F1 and B16F10 cells, and measured their activity on both receptors (Figure 2B). It was previously reported that active LRP-1 is derived from proteolytic cleavage of a ∼600 kDa precursor by the proprotein convertase furin into a ∼515 kDa extracellular domain and a ∼85 kDa TMD-containing C-terminal fragment, which are non-covalently linked [40]. In B16F1 cells, PCSK9 and its D374Y mutant reduce the levels of the ∼85 kDa fragment of LRP-1 by ∼40% and ∼50%, respectively. In contrast, no effect was observed on LDLR levels. In the more metastatic B16F10 cells, while both constructs reduced LRP-1 levels by ∼40% and ∼80%, respectively, only the PCSK9^D374Y^ mutant resulted in a ∼60% reduction of LDLR levels (Figure 2B). We conclude that in B16 melanoma cells PCSK9 enhances the degradation of LRP-1, and that the machinery to sort the [LDLR.PCSK9] and [LRP-1.PCSK9] complexes towards degradation compartments in B16F1 cells must be different.

### PCSK9 enhances the degradation of LRP-1 in HEK293 and HepG2 cells

**Figure 3:**
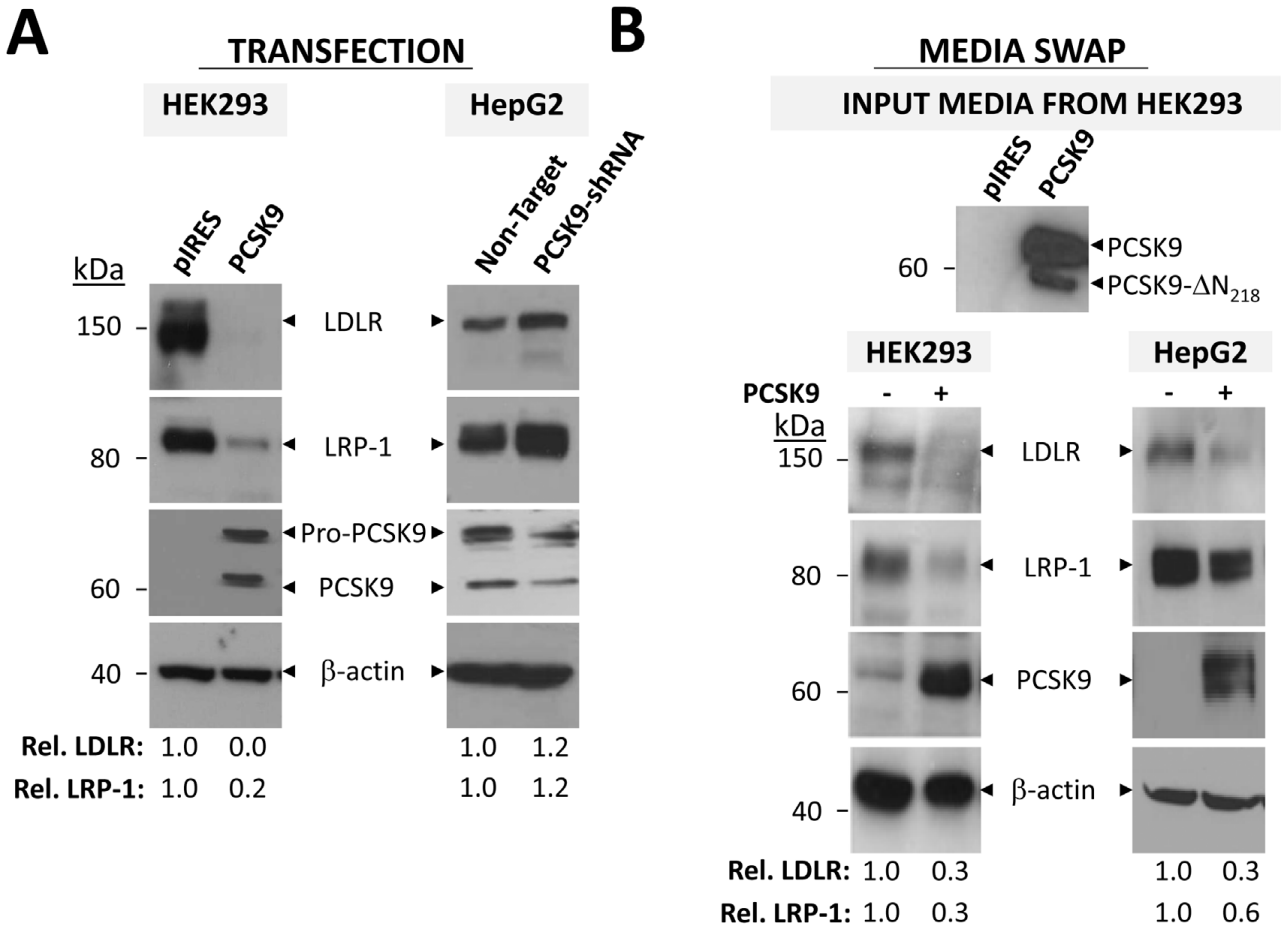
PCSK9 induces degradation of LRP-1. **A**) PCSK9 transfection. HEK293 cells were transfected with PCSK9-V5 or empty control pIRES-V5 vector prior to being lysed in 1x RIPA. LDLR and LRP-1 levels were examined by Western blot in these cells, as well as in HepG2 cells stably expressing PCSK9-shRNA. PCSK9 levels were assessed using mAb-V5 in HEK293 cells and an anti-PCSK9 antibody in HepG2 cells. The levels of LDLR and LRP-1 were estimated relative to β-actin. Data are representative of at least three independent experiments. **B**) PCSK9 media swap. Conditioned serum-free media collected from HEK293 cells transfected with PCSK9-V5 or empty control pIRES-V5 vector was collected and applied to naive HEK293 or HepG2 cells. The effect of exogenous PCSK9 on LDLR and LRP-1 was assessed by Western blotting with anti-hLDLR and LRP-1 antibodies respectively. Cell-associated PCSK9 was measured using mAb-V5. The relative intensities of LDLR and LRP-1 were normalized to β-actin using NIH ImageJ software. Data are representative of at least three independent experiments.

To assess whether PCSK9 is capable of inducing degradation of LRP-1 in other cell lines, the protein levels of LRP-1 were quantified in cellular extracts from HEK293 cells transfected with a control empty pIRES-V5 vector or one expressing PCSK9-V5 tagged. Western blotting revealed that compared to control, transfection of PCSK9 resulted in a ∼100% decrease in the levels of LDLR and a ∼80% decrease in ∼85 kDa LRP-1 (Figure 3A). Because the transfection efficiency of HepG2 cells is low, we examined LRP-1 levels in HepG2 cells stably expressing PCSK9-shRNA (with reduced endogenous PCSK9) as compared to HepG2 cells stably expressing a non-target shRNA [21]. The efficiency of the PCSK9 knockdown was determined using a rabbit anti-PCSK9 antibody and found to be ∼60% efficient. This decrease in PCSK9 resulted in a ∼20% increase in both LDLR and LRP-1 levels when normalized to β-actin (Figure 3A). Therefore, PCSK9 is capable of mediating the degradation of LRP-1 in both human HEK293 and HepG2 cells, as well as in mouse B16 melanoma cells.

To assess whether extracellular PCSK9 is capable of inducing degradation of LRP-1, HEK293 and HepG2 cells were incubated with pIRES-V5 or PCSK9-V5 conditioned media produced in HEK293 cells (Figure 3B). Naive HEK293 or HepG2 cells were incubated overnight with control or PCSK9 (∼0.7 μg/ml) conditioned media, at levels that previously resulted in a good response on LDLR [30,31[31], [32]. Western blot analyses of cell lysates showed that endogenous LRP-1 levels were diminished by ∼70% in HEK293 and ∼40% in HepG2 cells treated with 0.7 μg/ml exogenous PCSK9 [31], [32]. In similarly treated cells, LDLR levels were reduced by ∼70% of controls in both cell types (Figure 3B). Thus, PCSK9 is capable of inducing the degradation of LRP-1 in both HEK293 and HepG2 cells when transfected or added extracellularly.

To determine whether or not the ability of PCSK9 to mediate LRP-1 degradation, observed above (Figures 2B, 3A, and 3B), also occurs in the livers of *Pcsk9* ***^−/−^*** mice, the levels of total LRP-1 were compared to those of WT littermate mice (Figure S1A). No significant change was observed in LRP-1 protein levels between the two genotypes in both genders (Figure S1A), suggesting that PCSK9's ability to induce degradation of LRP-1 may be cell line specific. To further confirm the inability of PCSK9 to enhance the degradation of LRP-1 in liver, primary hepatocytes were isolated from the livers of WT mice and treated with PCSK9 conditioned media (Figure S1B). Addition of PCSK9 to WT hepatocytes did not induce any change in LRP-1. Thus the ability of PCSK9 to act on LRP-1 is a function of cell types, and for some reason it is unable to enhance the degradation of LRP-1 in either primary hepatocytes or whole liver.

### PCSK9 can degrade LRP-1 in the absence of the LDLR

**Figure 4:**
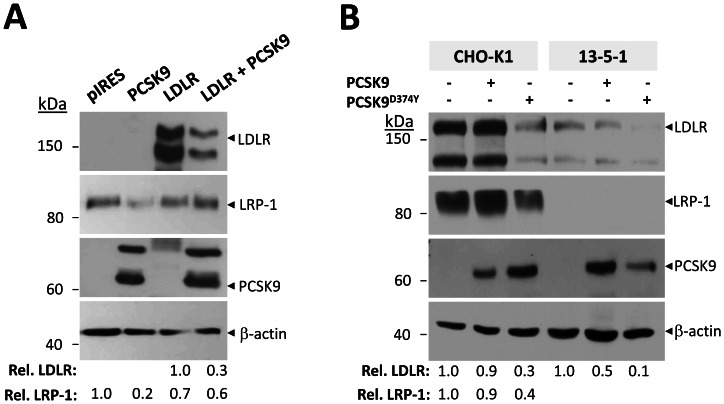
Independent actions of PCSK9 on the LDLR and LRP-1. **A**) PCSK9 acts on LRP-1 independent of the LDLR. CHO-A7 cells were transfected with control empty pIRES vector, PCSK9-V5, LDLR-V5, or both PCSK9-V5 and LDLR-V5. Levels of LRP-1 were measured 24 h after transfection by Western blotting using an anti-LRP-1 antibody. LDLR and PCSK9 were detected using mAb-V5. β-actin levels, as detected by an anti-β-actin antibody, were used to normalize the amounts of LDLR and LRP-1 quantified using NIH ImageJ software. Data are representative of two independent experiments. **B**) PCSK9 acts on the LDLR independent of LRP-1. CHO-K1 and CHO 13-5-1 were incubated overnight with conditioned serum-free media collected from HEK293 cells transfected with empty pIRES-V5 vector, PCSK9-V5, or PCSK9^D374Y^-V5. The cells were then lysed in 1x RIPA and submitted to Western blotting using the following antibodies: anti-hamster LDLR, anti-LRP-1, mAb-V5 to detect bound PCSK9, and anti-β-actin antibody. Intensities of the LDLR and LRP-1 were normalized to those of β-actin using NIH ImageJ software. Data are representative of three independent experiments.

To determine whether or not the effects of PCSK9 on LRP-1 are mediated through the LDLR, we examined the ability of PCSK9 to act on LRP-1 in CHO-A7 cells deficient in LDLR (Figure 4A). Accordingly, CHO-A7 cells were transfected with empty pIRES-V5 vector, PCSK9-V5, LDLR-V5, or both PCSK9-V5 and LDLR-V5. Twenty four-hours post-transfection, cell lysates were subjected to SDS-PAGE followed by Western blot analysis. The expression of PCSK9 alone resulted in a ∼80% decrease in LRP-1, suggesting that LDLR is not needed for the PCSK9-mediated LRP-1 degradation. However, when the LDLR was co-transfected with PCSK9 in CHO-A7 cells, only a ∼40% decrease in LRP-1 was observed, whereas LDLR was decreased by ∼70% (Figure 4A). Thus, for similar PCSK9 and LRP-1 protein levels (Figure 4A), the presence of LDLR reduces the ability of PCSK9 to enhance the degradation of LRP-1 from ∼80% to ∼40%, indicating a competition between LDLR and LRP-1 for PCSK9. Finally, transfection of the LDLR alone resulted in ∼30% lower levels of endogenous LRP-1, suggesting the existence of a balance between LRP-1 and LDLR levels at the protein level, independent of PCSK9.

Similarly, to determine whether or not the effects of PCSK9 on the LDLR require LRP-1, and whether LRP-1 is a critical co-regulator of the PCSK9-induced degradation of the LDLR, CHO 13-5-1 cells, which lack endogenous LRP-1 [41], were treated with pIRES-V5, PCSK9-V5 or PCSK9^D374Y^-V5 conditioned media produced from HEK293 cells (Figure 4B). CHO 13-5-1 cells were compared to similarly treated parental CHO-K1 cells, which express LRP-1 endogenously [41]. Incubation of CHO 13-5-1 or CHO-K1 cells with ∼0.7 μg/ml PCSK9 [31], [32] resulted in a ∼50% and ∼10% decrease in LDLR, respectively, whereas treatment with GOF PCSK9^D374Y^ caused a ∼90% and ∼70% decrease in LDLR levels when normalized to β-actin, respectively (Figure 4B). We also note that the GOF PCSK9^D374Y^ is more active on both LDLR and LRP-1. In conclusion, while PCSK9 can enhance the degradation of LRP-1, the latter is not a critical co-factor in the PCSK9-mediated degradation of the LDLR.

To identify other receptors that may be critical in the ability of PCSK9 to induce LDLR degradation, quantitative proteomic analysis was performed on the membrane fractions isolated by centrifugation from the livers of 3 month old littermate WT and *Pcsk9* ***^−/−^*** mice in a pure C57BL/6 background [12], [42] (*see* Experimental Procedures). Proteins listed in Table 1 are those identified by mass spectrometry and filtered according to the following criteria: significant differences (p≤0.05) between WT and *Pcsk9* ***^−/−^*** livers, membrane-bound secretory proteins that contain a signal peptide/membrane anchor, not localized exclusively to the ER or mitochondria, and exhibiting at least one TMD. The “Ratio” values indicate the relative abundance of the protein in *Pcsk9* ***^−/−^*** *versus* WT livers. The LDLR was found to be the most upregulated (∼2.5×, p<0.01) protein in *Pcsk9* ***^−/−^*** livers, as previously reported [11], [12]. Notably, no other membrane-bound protein was found to increase (or decrease) to a similar extent. If the postulated LDLR co-receptor is recycled rather than degraded, it is possible that its levels would not be significantly altered by the absence of PCSK9. Looking for proteins with at least one **NP**X**Y** motif in their CT led us to identify the EGFR (Table 1). EGFR levels were found to be slightly decreased (∼15%, p<0.01) in the livers of *Pcsk9* ***^−/−^*** mice (Table 1), although PCSK9 did not induce degradation of the EGFR in HuH7 cells (Figure S2).

**Table 1:** Proteomics analysis of the livers of WT *versus Pcsk9^−/−^* mice.

UPREGULATED			DOWNREGULATED								
*GENE*	*Ratio*	*P*	*GENE*	*Ratio*	*P*	*GENE*	*Ratio*	*P*	*GENE*	*Ratio*	*P*
***Ldlr***	***2.47***	***<0.01***	Bsg	0.94	0.02	***Lrp1***	***0.79***	***<0.01***	Hpx	0.58	<0.01
Ugt2a3	1.74	<0.01	Itgb1	0.94	0.03	Man1a	0.78	<0.01	Gltpd2	0.58	<0.01
Ugt1a2	1.51	<0.01	Alcam	0.94	0.03	Lman1	0.77	<0.01	Dhrs7b	0.55	<0.01
Ugt1a2	1.51	<0.01	Itfg3	0.93	0.01	Pigr	0.77	<0.01	Icam1	0.55	<0.01
Cyp2c44	1.48	<0.01	Cml2	0.91	0.01	Gaa	0.76	0.01	St3gal4	0.49	<0.01
Cyp2d22	1.37	0.01	Abcc2	0.90	0.01	Acbd5	0.75	<0.01			
Ugt2b1	1.32	<0.01	Hsd17b13	0.89	<0.01	Abca6	0.75	0.03			
Ugt2b36	1.32	<0.01	Col4a2	0.88	0.05	Glg1	0.72	0.02			
Hsd11b1	1.32	0.01	Gjb1	0.87	0.02	Tm9sf2	0.72	0.02			
Cisd1	1.27	<0.01	Anpep	0.87	<0.01	St6gal1	0.72	<0.01			
Lass2	1.24	0.03	B2m	0.85	<0.01	Slc22a7	0.71	<0.01			
Cyp2e1	1.20	0.01	Atp1b1	0.85	0.02	Man1b1	0.71	<0.01			
Hsd17b13	1.20	<0.01	***Egfr***	***0.85***	***<0.01***	Scarb1	0.68	<0.01			
Ugt2b34	1.19	0.03	Lman2	0.84	<0.01	Tm9sf4	0.67	<0.01			
Slc27a5	1.18	<0.01	C3	0.84	0.03	Tm9sf3	0.65	<0.01			
Apmap	1.15	0.02	Tmed10	0.84	0.02	M6pr	0.62	<0.01			
Skint5	1.14	<0.01	Scarb2	0.83	<0.01	Tmed7	0.62	<0.01			
Slc2a2	1.12	0.04	Ces2	0.81	0.01	Mgat2	0.62	<0.01			
Enpep	1.09	0.01	Dsg2	0.80	<0.01	Gpr56	0.60	<0.01			
Fam176a	1.06	0.04	Man2a1	0.79	0.03	Slc35a2	0.59	0.01			

Proteomics analysis of the livers of *Pcsk9^−/−^* mice. List of membrane proteins identified by quantitative mass spectrometry of livers isolated from *Pcsk9^−/−^* mice and compared to their WT littermates. The proteins listed here include only membrane-bound secretory proteins containing signal peptide/membrane anchors, TMDs, and not exclusively localized in the ER or mitochondrial compartments. “Ratio” values indicate the relate abundance of the protein in *Pcsk9^−/−^* livers *versus* those of WT mice.

### The catalytic domain of PCSK9 is required for degradation of LRP-1

**Figure 5:**
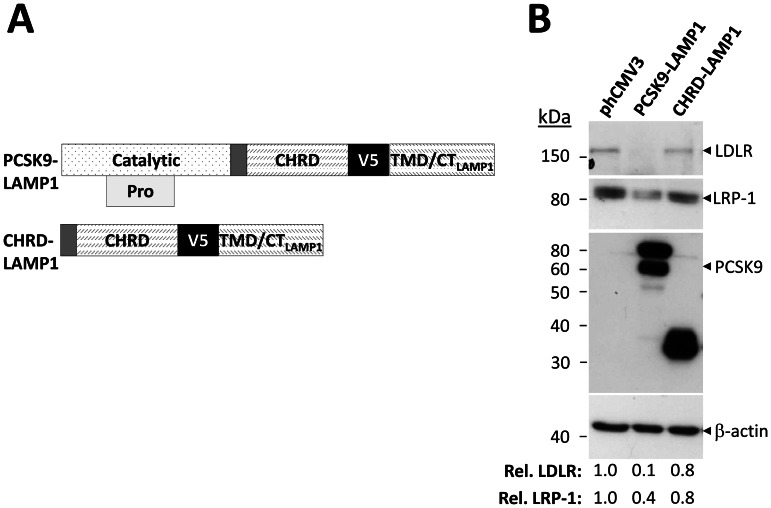
The catalytic domain of PCSK9 is required for LRP-1 degradation. **A**) Chimeric PCSK9 constructs. Schematic representation of PCSK9 and its CHRD coupled to the TMD and CT of LAMP1. The constructs contained C-terminal V5-tags. **B**) HEK293 cells were transfected with PCSK9 and the CHRD-LAMP1 chimeric constructs. Expression of the PCSK9 constructs was assessed by Western blotting using mAb-V5. The effects of these constructs on the levels LDLR and LRP-1 were determined by immunoblotting with anti-human LDLR and LRP-1 antibodies. LDLR and LRP-1 levels were measured relative to β-actin levels. Data are representative of at least three independent experiments.

While the catalytic domain (aa 153–407) of PCSK9 is required for its binding to the LDLR, it is not sufficient to induce degradation of this receptor [17], requiring the CHRD for that purpose [10]. To compare the domain(s) of PCSK9 required for degradation of LRP-1 *versus* LDLR, we transfected HEK293 cells with a PCSK9 chimera lacking its catalytic domain (Figure 5A). The chimeric secretory CHRD-LAMP1 construct contained the CHRD of PCSK9, as well as the TMD and CT of LAMP1, as described [10], [34]. The TMD-CT segment of LAMP1 directs sorting to endosomal/lysosomal compartments, and when fused to domains of PCSK9 results in enhanced sorting of PCSK9 targets (e.g., LDLR, VLDLR and ApoER2) to the degradation pathway [10], [34]. The effect of these constructs on LRP-1 levels was examined by immunoblotting (Figure 5B).

PCSK9-LAMP1 induced degradation of both LRP-1 (∼60% decrease) and LDLR (∼90% decrease). In contrast, the CHRD-LAMP1 construct had no significant effect (∼20% reduction) on either LRP-1 or LDLR levels, suggesting that the CHRD is insufficient to induce degradation of either receptor. Thus, the catalytic domain of PCSK9 is required to effectively induce degradation of LRP-1, as was originally observed for LDLR and its closest family members VLDLR and ApoER2 [34]. In conclusion, the structural requirements for the catalytic domain of PCSK9 to induce the degradation of LRP-1 are similar to those needed to enhance the degradation of the LDLR and other family members.

## Discussion

The adaptor protein ARH that binds **NP**X**Y** motifs [24] has been demonstrated to be essential in the PCSK9-dependent degradation of the LDLR [23]. The cell surface protein that binds ARH and hence regulates the [LDLR.PCSK9] endocytosis was originally thought to be the LDLR itself. However, the CT and TMD of the LDLR are not essential in mediating PCSK9-enhanced degradation (Figure 1), suggesting the presence of an additional factor(s) at the cell surface, which interacts with ARH through an **NP**X**Y** motif. To identify and investigate novel partners implicated in the PCSK9-regulated degradation of the LDLR, we looked for membrane-bound proteins that contain an **NP**X**Y** motif. LRP-1, a member of the LDLR family, contains two **NP**X**Y** motifs in its CT, is a type I membrane-bound endocytic receptor that is processed by a furin-like convertase(s) to form a short C-terminal (∼85 kDa) and a large N-terminal (∼515 kDa) subunit that binds to all known LRP ligands [37], [43], [44]. While we herein show that LRP-1 is a novel PCSK9 target in HEK293 and HepG2 cells (Figure 3), it is not the sought co-factor. This is based on the fact that its presence is not essential for PCSK9 to degrade cellular LDLR (Figure 4B) and *vice versa* (Figure 4A). This finding in HepG2 cells lacking PCSK9 differs from what we previously observed in HepG2 cells stably expressing PCSK9 at low levels where no appreciable effect was seen on LRP-1 [8]. This difference in LRP-1 level may be due to insufficient overexpression of PCSK9 in the HepG2 cells previously examined [8].

Even though LRP-1 is not the sought co-factor that is needed for the sorting of the [LDLR.PCSK9] complex to endosomes/lysosomes, it is a novel target of PCSK9. Upon examining the ability of PCSK9 to act on LRP-1 in B16F1/F10 melanoma cells (Figure 2), we discovered that PCSK9 is capable of enhancing LRP-1 degradation in both the B16F1 and the more metastatic B16F10 cells, but only the more active GOF PCSK9^D374Y^ is capable of acting on the LDLR in B16F10, and not in B16F1 cells. Because this is one instance where LRP-1 degradation by PCSK9 is favoured over LDLR, we conclude that in B16 melanoma cells the machinery to sort the [LDLR.PCSK9] and [LRP-1.PCSK9] complexes towards degradation compartments is different. Furthermore, we showed that PCSK9 can enhance the degradation of LRP-1 in various cell lines (Figures 3 and 4). Whether this applies to other types of cells and/or tissues is yet to be investigated.

For LDLR [6], [19], and its closest family members VLDLR and ApoER2 [34], the catalytic domain of PCSK9 is necessary to bind the EGF-A domain of these receptors, but is not sufficient to induce their degradation. While the requirement of the integrity of the catalytic domain of PCSK9 to induce the degradation of LRP-1 and LDLR is similar (Figure 5), the critical PCSK9 residues seem to be somewhat different. While both the LDLR and LRP-1 contain similar EGF-A domains, the sequence identity of this domain between the two receptors is ∼48% (Figure S3). The crucial PCSK9 binding residues in LDLR's EGF-A domain NECL_319_ and D_331_ are found in equivalent positions in LRP-1 [18]. However, the critical H_327_ and Y_336_ in LDLR are replaced by Q_2954_ and F_2963_ in LRP-1, respectively (Figure S3). The binding site in the catalytic domain of PCSK9 contains D_374_ that forms a hydrogen bond with H_327_ of the LDLR [16]. This was the rationale behind the GOF PCSK9^D374Y^ mutant, as it binds ∼25-fold better the LDLR. In that context, the crystal structure of the [PCSK9.EGF-A^H327Y^] complex shows that replacement of H_327_ by Y_327_ results in a strong hydrogen bond with D_374_ in PCSK9 at neutral pH. In LRP-1, the H_327_ is replaced by Q_2954_ (Figure S3). Whether sequence substitutions of H_327_ and Y_336_ in the EGF-A domain of LDLR (Figure S3) into the Q_2954_ and F_2963_ substitutions in LRP-1 result in a lower affinity for PCSK9, as predicted from our results (Figures 2 and 3), is not known, but may in part explain the lower efficacy of PCSK9 to enhance the degradation of LRP-1 compared to LDLR in most cell types, except for melanoma B16 cells.

In order to begin to identify tissues where LRP-1 would be sensitive to PCSK9, we compared by Western blot the levels of ∼85 kDa LRP-1 liver from WT and *Pcsk9* ***^−/−^*** mice. Even though LDLR levels were increased in knockout mice, we did not detect significant differences in LRP-1 levels in mouse liver (males and females; Figure S1A). In this context, it was originally found that in cells, PCSK9 enhances the degradation of the LDLR family members VLDLR and ApoER2 [34], and yet no effect was detected by Western blot of these receptors in livers from *Pcsk9* ***^−/−^*** mice [42]. However, later on it was found that VLDLR is a PCSK9 target in female adipose tissue (but much less so in males) [42], and that ApoER2 is degraded by PCSK9 in brain-derived cerebellar granule neurons [45]. Additionally, Annexin A2 is an endogenous inhibitor of PCSK9 activity on LDLR [46], and it was also found that WT adrenals are refractory to PCSK9 activity, but LDLR levels in the adrenals of mice lacking Annexin A2 are sensitive to PCSK9 [47]. Whether liver also expresses a protein that prevents the function of PCSK9 on LRP-1 is still unclear. Nevertheless, it is noteworthy that the mammalian ATP-binding cassette transporter ABCA7, which is abundant in liver, was reported to co-localize and enhance the stability of LRP-1 at the cell surface [48](P. Fraser, U. Toronto, *personal communication*), and may thus prevent the function of PCSK9 on LRP-1 in this tissue. It is also possible that differential levels of ABCA7 or another protein may limit the ability of PCSK9 to degrade LRP-1 in certain cell lines. It will thus be of importance in the future to discover where LRP-1 is a target for PCSK9.

In conclusion, while PCSK9 can enhance the degradation of LRP-1, the latter is not an essential factor for LDLR regulation, but LDLR can effectively compete with LRP-1 for PCSK9 activity. Reduction of circulating PCSK9 levels or activity is presently an attractive approach to lower LDLc and associated risk of developing cardiovascular disease [13], [49]. The observation presented here that LRP-1 protein levels could also be regulated by PCSK9 is significant, since PCSK9 was recently shown to modulate the metastatic potential of melanoma cells [29] and LRP-1 is a well-known factor involved in tumour metastasis [38], [50].

## Supporting Information

Figure S1 **Comparison of LRP-1 in the livers of WT and** ***Pcsk9^−/−^*** **mice and effect of PCSK9 on WT primary hepatocytes.** **A**) LRP-1 levels in the livers of male and female WT and *Pcsk9^−/−^* mice [12] were assessed by Western blotting. Tissues were lysed in 1x RIPA and submitted to Western blotting using anti-mouse LDLR, anti-LRP-1, and anti-β-actin antibodies. The intensities of the LDLR and LRP-1 were normalized to those of β-actin using ImageJ. Data are representative of two independent experiments and tissue derived from at least five mice. **B**) Primary hepatocytes were isolated from the livers of WT mice. Twenty-four hours after isolation, the primary hepatocytes were treated overnight with pIRES-V5 or PCSK9-V5 conditioned media produced in HEK293 cells. Western blot analysis was performed on lysates from the primary hepatocytes and levels of LRP-1 and LDLR examined using anti-LRP-1 and anti-mLDLR antibodies respectively. LRP-1 and LDLR intensities were normalized to those of β-actin.
(TIF)

Figure S2 **PCSK9 does not induces degradation of the EGFR in HuH7 cells.** HuH7 cells were transfected with PCSK9-V5 or empty control pIRES-V5 vector prior to being lysed in 1× RIPA. Endogenous LDLR and EGFR levels were examined by Western blot in these cells. PCSK9 levels were assessed using a mAb-V5. The levels of LDLR and EGFR were estimated relative to β-actin. Data are representative of two independent experiments.
(TIF)

Figure S3 **Amino acid sequence alignment of the EGF-A domains of LDLR and LRP-1.** The EGF-A domain of the LDLR was aligned with the most similar EGF domain found in LRP-1. While residues that are identical in the two domains are shown in bold, those in the LDLR which have previously been demonstrated to be critical in the interaction with PCSK9 are shown in red. Bold and underlined residues, F_2963_ in LRP-1 (equivalent to Y_336_ in LDLR) and H_327_ in the LDLR (replaced by Q_2954_ in LRP-1), are critical residues which differ between the two receptors.
(TIF)
