# Supplementary figures and images for: Proprotein Convertase Subtilisin/Kexin Type 9 (PCSK9) Can Mediate Degradation of the Low Density Lipoprotein Receptor-Related Protein 1 (LRP-1)
Source: PLoS One. 2013 May 13;8(5):e64145. doi: 10.1371/journal.pone.0064145 (PMC3652815; doi:10.1371/journal.pone.0064145)

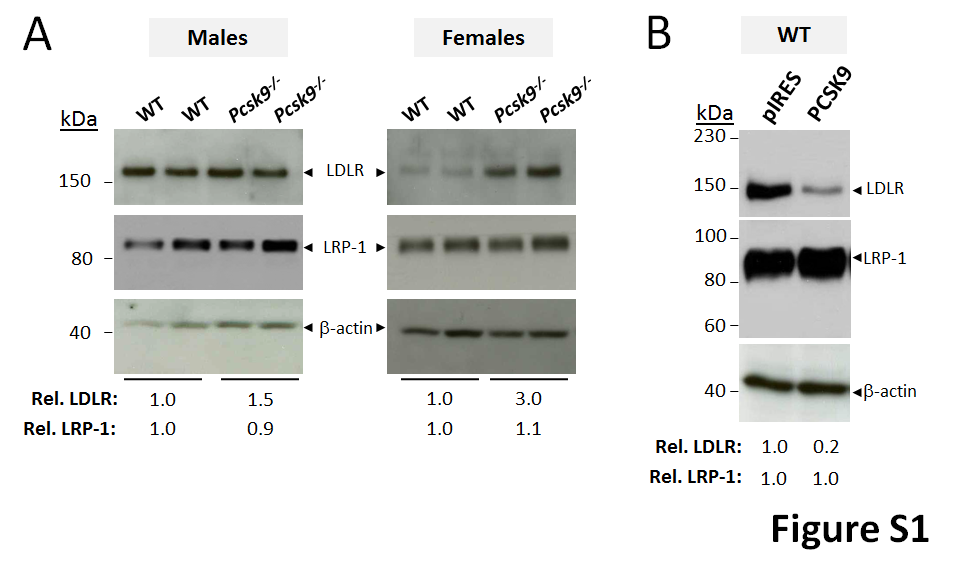

Supplement: Figure S1 — Comparison of LRP-1 in the livers of WT and Pcsk9−/− mice and effect of PCSK9 on WT primary hepatocytes. A) LRP-1 levels in the livers of male and female WT and Pcsk9−/− mice [12] were assessed by Western blotting. Tissues were lysed in 1x RIPA and submitted to Western blotting using anti-mouse LDLR, anti-LRP-1, and anti-β-actin antibodies. The intensities of the LDLR and LRP-1 were normalized to those of β-actin using ImageJ. Data are representative of two independent experiments and tissue derived from at least five mice. B) Primary hepatocytes were isolated from the livers of WT mice. Twenty-four hours after isolation, the primary hepatocytes were treated overnight with pIRES-V5 or PCSK9-V5 conditioned media produced in HEK293 cells. Western blot analysis was performed on lysates from the primary hepatocytes and levels of LRP-1 and LDLR examined using anti-LRP-1 and anti-mLDLR antibodies respectively. LRP-1 and LDLR intensities were normalized to those of β-actin. (TIF) [file pone.0064145.s001.tif]

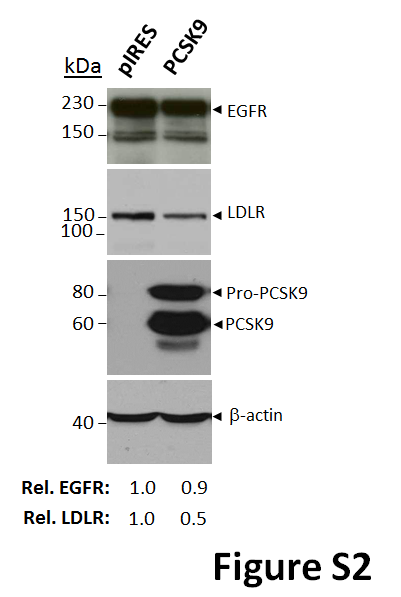

Supplement: Figure S2 — PCSK9 does not induces degradation of the EGFR in HuH7 cells. HuH7 cells were transfected with PCSK9-V5 or empty control pIRES-V5 vector prior to being lysed in 1× RIPA. Endogenous LDLR and EGFR levels were examined by Western blot in these cells. PCSK9 levels were assessed using a mAb-V5. The levels of LDLR and EGFR were estimated relative to β-actin. Data are representative of two independent experiments. (TIF) [file pone.0064145.s002.tif]

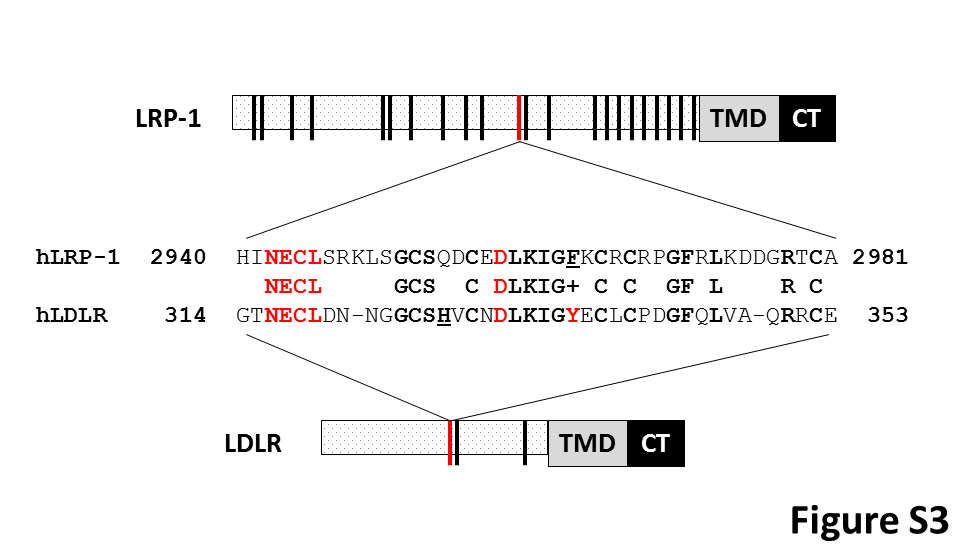

Supplement: Figure S3 — Amino acid sequence alignment of the EGF-A domains of LDLR and LRP-1. The EGF-A domain of the LDLR was aligned with the most similar EGF domain found in LRP-1. While residues that are identical in the two domains are shown in bold, those in the LDLR which have previously been demonstrated to be critical in the interaction with PCSK9 are shown in red. Bold and underlined residues, F 2963 in LRP-1 (equivalent to Y336 in LDLR) and H 327 in the LDLR (replaced by Q2954 in LRP-1), are critical residues which differ between the two receptors. (TIF) [file pone.0064145.s003.tif]
